# Expression of *EPL1* from *Trichoderma atroviride* in *Arabidopsis* Confers Resistance to Bacterial and Fungal Pathogens

**DOI:** 10.3390/plants12132443

**Published:** 2023-06-25

**Authors:** Mónica Montserrat Rojas Moreno, Enrique González-Pérez, Aida Araceli Rodríguez-Hernandez, María Azucena Ortega-Amaro, Alicia Becerra-Flora, Mario Serrano, Juan Francisco Jiménez-Bremont

**Affiliations:** 1 Laboratorio de Biotecnología Molecular de Plantas, División de Biología Molecular, Instituto Potosino de Investigación Científica y Tecnológica A.C., San Luis Potosí 78216, Mexico; 2 CONAHCyT-Instituto Politécnico Nacional, CEPROBI, Km. 6.5 Carr. Yautepec-Jojutla Col. San Isidro, Calle CEPROBI No. 8, Yautepec 62739, Mexico; 3 Coordinación Académica Región Altiplano Oeste, Universidad Autónoma de San Luis Potosí, Salinas de Hidalgo 78290, Mexico; 4 Centro de Ciencias Genómicas, Universidad Nacional Autónoma de México, Cuernavaca 62210, Mexico

**Keywords:** *Arabidopsis thaliana*, *Botrytis cinerea*, cerato-platanin proteins, elicitor, *Pseudomonas syringae*, *Trichoderma atroviride*

## Abstract

During plant interaction with beneficial microorganisms, fungi secrete a battery of elicitors that trigger plant defenses against pathogenic microorganisms. Among the elicitor molecules secreted by Trichoderma are cerato-platanin proteins, such as EPL1, from *Trichoderma atroviride*. In this study, *Arabidopsis thaliana* plants that express the *TaEPL1* gene were challenged with phytopathogens to evaluate whether expression of EPL1 confers increased resistance to the bacterial pathogen *Pseudomonas syringae* and the necrotrophic fungus *Botrytis cinerea*. Infection assays showed that *Arabidopsis EPL1-2*, *EPL1-3*, *EPL1-4* expressing lines were more resistant to both pathogens in comparison to WT plants. After *Pseudomonas syringae* infection, there were reduced disease symptoms (e.g., small chlorotic spots) and low bacterial titers in the three *35S::TaEPL1* expression lines. Similarly; *35S::TaEPL1* expression lines were more resistant to *Botrytis cinerea* infection, showing smaller lesion size in comparison to WT. Interestingly, an increase in ROS levels was detected in *35S::TaEPL1* expression lines when compared to WT. A higher expression of SA- and JA-response genes occurred in the *35S::TaEPL1* lines, which could explain the resistance of these EPL1 expression lines to both pathogens. We propose that EPL1 is an excellent elicitor, which can be used to generate crops with improved resistance to broad-spectrum diseases.

## 1. Introduction

The genus *Trichoderma* comprises many rhizocompetent filamentous fungi found in different ecosystems [1]. *Trichoderma* species are fast-growing, opportunistic invaders and prolific producers of secondary metabolites with antibiotic activity that suppresses diverse phytopathogens such as bacteria, fungi, and nematodes [2,3,4]. These properties make these fungi an ecologically dominant species. Other biocontrol mechanisms used by Trichoderma include mycoparasitism and competition for space and nutrients [5]. Due to the ability of *Trichoderma* spp. to suppress different phytopathogenic fungi, some species have been widely used in agriculture as biological control agents [6,7].

Some *Trichoderma* species have the ability to establish symbiotic relationships with plants. This symbiosis is achieved through crosstalk, in which plants and Trichoderma produce a wide range of chemical compounds that modify the transcriptomes, proteomes, and metabolomes of host plants [8,9]. As a result, *Trichoderma* species produce a wide range of compounds such as elicitors, siderophores, phytohormones, and volatile organic compounds (VOCs) that modulate plant growth and defense [10,11,12]. Elicitors are molecules that originate from the host plant (endogenous elicitors) or from the microbe (exogenous elicitors) and can induce biochemical and/or structural responses associated with the expression of resistance to plant diseases [13]. Among the elicitors produced by Trichoderma are the cerato-platanin proteins, which can activate the plant defense system against different kinds of phytopathogens [14].

Cerato-platanins (CPs) are proteins secreted by fungi that participate in diverse stages of the host-fungus interaction, acting as virulence factors or elicitors [15]. These CPs are composed of small cysteine-rich proteins of approximately 100 to 130 residues, and they have a signal peptide for their secretion [16]. The first CPs elicitors studied in Trichoderma were SM1 from *T. virens* and Eliciting Protein-Like (EPL1) from *T. atroviride* [14,17]. Exogenous application of SM1 purified from *T. virens* [14] as well as recombinant SM1 generated in the yeast *Pichia pastoris* [18] increased the expression of both local and systemic defense-related genes in cotton and maize plants. It was shown that the exogenous application of purified SM1 on cotton cotyledons reduced the size of the lesion caused by the pathogenic fungus *Colletotrichum* sp. [14]. In addition, maize and tomato plants inoculated with *T. virens* Gv29-8 that overexpressed *SM1* were more resistant to necrotrophic and biotrophic phytopathogens, respectively [19,20].

The Eliciting Plant Response-Like 1 (Epl1) elicitor is produced and exported by *T. atroviride* to the plant, and it is important in the interaction of fungi with plants because it stimulates the induction of defense responses in the plant [20,21]. *T. atroviride* strains (TaOE) that overexpress the *EPL1* gene increase disease resistance against different phytopathogens in tomato plants, whereas *Epl1* knockout mutants conferred less protection in tomato plants against *Alternaria solani* and *B. cinerea* [20]. While there is evidence that the EPL1 elicitor of *T. atroviride* activates the plant’s defense system, to the best of our knowledge, there are no reports on the molecular mechanism of this elicitor in plants.

The exogenous application of Epl1 protein from *T. asperellum* strain T4 (expressed and purified from *Pichia pastoris*) to soybean leaves showed protective activity against the pathogen *Cercosporidium sofinum* [22]. A similar study showed that the application of *EPL1* protein of *T. asperellum* also produced in *P. pastoris* significantly reduced the infection of Populus plantlets by *A. alternata* [23].

Other fungal CPs have been shown to induce resistance in plants by exogenous treatments. In the fungus *Botrytis cinerea*, CP BcSpl1 is one of the most abundant proteins in the secretome of this plant pathogen. Knockout mutants of *bcspl1* showed a reduction in virulence on hosts [24]. It was shown that the exogenous application of the BcSpl1 protein by infiltration in tobacco leaves induces resistance against *Pseudomonas syringae* and *B. cinerea*, which correlated with the induction of the PR-1α and PR-5 genes [25]. In addition, the CP Pop1 secreted by *Ceratocystis populicola*, a poplar pathogen, has been shown to induce phytoalexin synthesis, production of ROS and NO, and the induction of defense related genes in *Platanus acerifolia* leaves [15].

The heterologous expression of the SM1 elicitor of the phytopathogenic fungus *Magnaporthe oryzae* in *Arabidopsis* and rice plants has also been reported. Transgenic lines of *Arabidopsis* and rice that express SM1 have a greater resistance against fungal and bacterial diseases [26,27]. However, transient expression of MoSM1 in rice leaves or high expression levels of the MoSM1 gene in transgenic lines of *Arabidopsis* generated HR-like necrosis. This could be due to the fact that some CPs of phytopathogenic fungi, such as CP from *Ceratocystis fimbriata f. sp. platani* [28], BcSpl1 from *B. cinerea* [24], and MoSM1 or MoMSP1 from *M. oryzae*, cause phytotoxic effects in different plants [26,29].

To determine if the SM1 homolog of a symbiotic fungus expressed in *Arabidopsis* confers disease resistance but without producing the toxic effect on the plant, we generated *Arabidopsis thaliana* plants that express the *EPL1* gene from *T. atroviride*. These *Arabidopsis TaEPL1* expressing lines were infected with the bacterium *P. syringae* and the necrotrophic fungus *B. cinerea*. We achieved a higher resistance against these pathogens in plants expressing *TaEPL1*. We also observed high levels of hydrogen peroxide accumulation in *TaEPL1* expressing lines compared to WT plants. Expression analysis of plant hormone-related genes showed that the transgenic *EPL1* lines induced SA and JA related genes, which could explain the enhanced resistance of these transgenic lines to the tested pathogens. 

## 2. Results

### 2.1. Generation of Arabidopsis EPL1 Expressing Lines (35S::TaEPL1)

We generated *Arabidopsis* transgenic plants expressing the *EPL1* elicitor gene from the beneficial fungus *T. atroviride* (*TaEPL1*). The *35S::TaEPL1* construct (Supplementary Figure S1A) was transformed by floral dip in *A. thaliana* ecotype Col-0 (WT). The *EPL1* gene expression of three *35S::TaEPL1* lines (*EPL1-2*, *EPL1-3* and *EPL1-4*) was quantified by RT-qPCR in 15-day-old plants. The highest *EPL1* mRNA expression level was found in the *EPL1-3* line, followed by the *EPL1-4* and *EPL1-2* expression lines. As expected, *EPL1* transcript was not detected in the *Arabidopsis* WT plants (Supplementary Figure S1B). 

### 2.2. The Expression of the EPL1 Gene in Arabidopsis Results in Accelerated Growth

**Figure 1:**
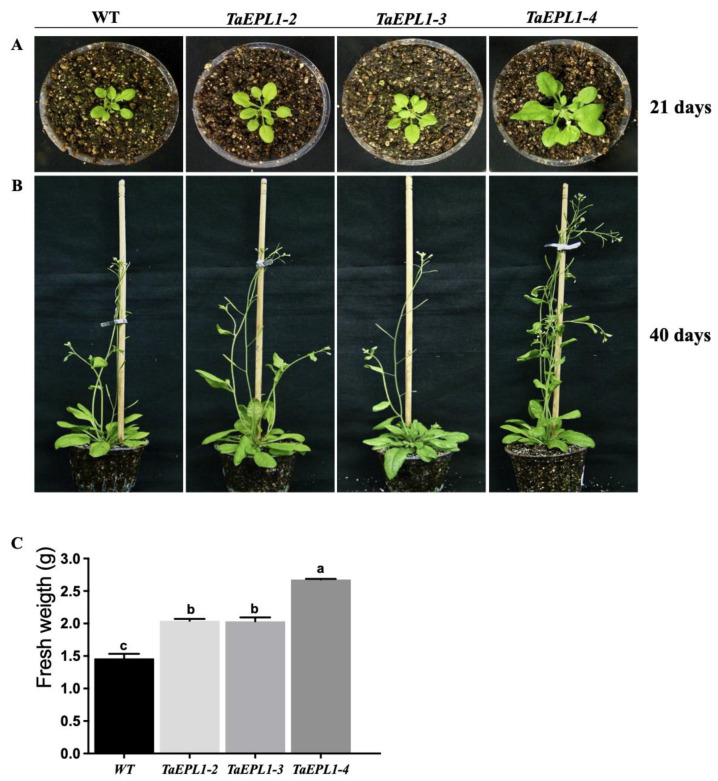
Phenotype of Arabidopsis *35S::TaEPL1* expressing lines. Photographs of Arabidopsis plants grown on soil pots under long day conditions, showing the WT, *35S::EPL1-2*, *35S::EPL1-3*, and *35S::EPL1-4* lines at two different stages: 21-day-old (**A**) and 40-day-old (**B**). Data on fresh weight (g) of the leaf area in 40-day-old plants for the following lines: WT, *35S::EPL1-2, 35S::EPL1-3*, and *35S::EPL1-4* (**C**). Data are means ±SE (*n* = 6). Statistical analysis between genotypes was determined by One-way ANOVA, and the letters indicate statistically significant differences by Tukey’s test at *p* < 0.05.

The expression of the Trichoderma *EPL1* gene in Arabidopsis *(35S::TaEPL1 lines)* resulted in a positive impact on its growth, as observed in 21-day-old plants grown in soil (Figure 1A). In particular, the *35S::TaEPL1-4* line displayed the highest development among the three *TaEPL1* expressing lines (Figure 1A). As previously observed, 40-day-old plants of the *35S::TaEPL1-4* exhibited the most significant development, while the parental Col-0 (WT) displayed the lowest growth (Figure 1B). We evaluated the fresh weight of the aerial parts of each 40-day-old plant, and all three *TaEpl1* expressing lines exhibited higher fresh weights than the WT. Among them, the *35S::TaEpl1-4* line exhibited the highest biomass, as evidenced by a greater number of developed inflorescences (Figure 1B,C). 

### 2.3. The Arabidopsis 35S::TaEPL1 Lines Are More Resistant to Infection by Pseudomonas Syringae

**Figure 2:**
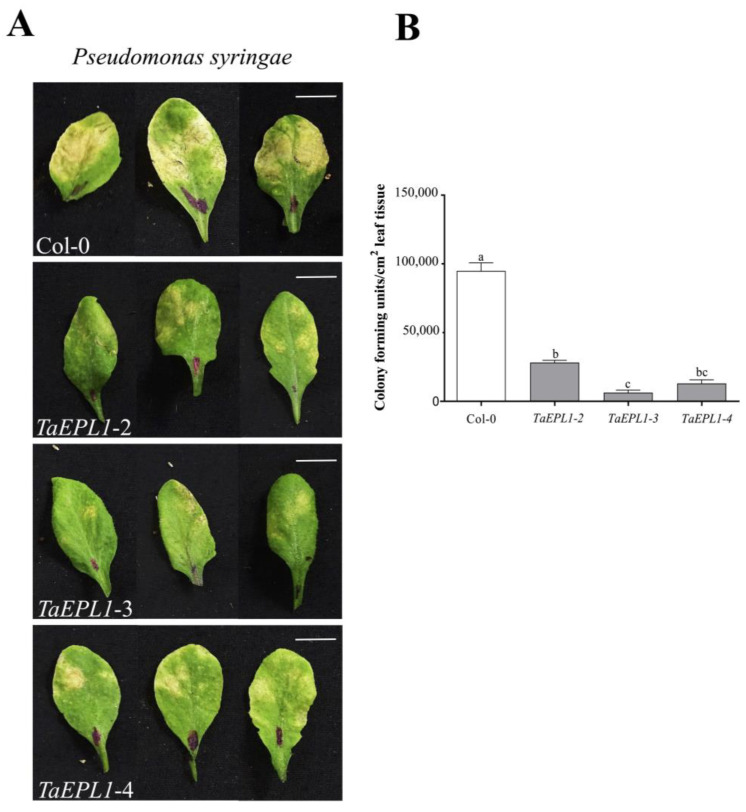
*Pseudomonas syringae* infection of *Arabidopsis 35S::TaEPL1* and WT leaves. (**A**) Disease symptoms in leaves of four-week-old *35S::TaEPL1* (*TaEPL1-2*, *TaEPL1-3* and *TaEPL1-4*) and WT plants 72 h after infection with *P. syringae* DC3000 strain. In the images, the scale bar indicates 1 cm. (**B**) To determine colony-forming units (CFU), five independent 0.5 cm^2^ leaf disks from *35S::TaEPL1* and WT plants were ground, and then the ground leaves extract was spotted onto LB plates with rifampicin to count the bacterial colonies formed. Data represented graphically. The bars denote the ±SE from five biological replicates (*n* = 5). Statistical analysis between genotypes was determined by One-way ANOVA, and the letters indicate statistically significant differences by Tukey’s test at *p* < 0.05.

The *Arabidopsis TaEPL1* expression lines were inoculated with *P. syringae* pv. tomato DC3000 strain (*Pst*) to examine whether the expression of the *TaEPL1* elicitor confers resistance against bacterial infection. Four-week-old *Arabidopsis EPL1-2*, *EPL1-3*, *EPL1-4*, and WT plants were inoculated with *Pst* bacterial suspension. Disease symptoms and bacteria colony-forming units were recorded after 72 hpi (Figure 2). We observed more severe symptoms with larger chlorotic spots in the WT plants than the *35S::TaEPL1* expression lines (Figure 2A). This is in agreement with the low levels of bacterial titers detected in the *Arabidopsis 35S::TaEPL1* expression lines, which were up to 14.8-fold lower than that obtained in the WT plants (Figure 2B). The *EPL1-3* line presented the lowest bacterial titers (6.3 × 10^3^) at 72 hpi and showed milder symptoms of *Pst* infection in comparison to the other two overexpressing lines (Figure 2B). This correlates with the *EPL1-3* line having the highest *EPL1* transcript levels among the transgenic lines (Supplementary Figure S1). Our data reveal that expression of the *EPL1* gene from the beneficial fungus Trichoderma in *Arabidopsis* plants confers marked resistance to *P. syringae* infection.

### 2.4. The Arabidopsis 35S::TaEPL1 Lines Display Increased Reactive Oxygen Species (ROS)

**Figure 3:**
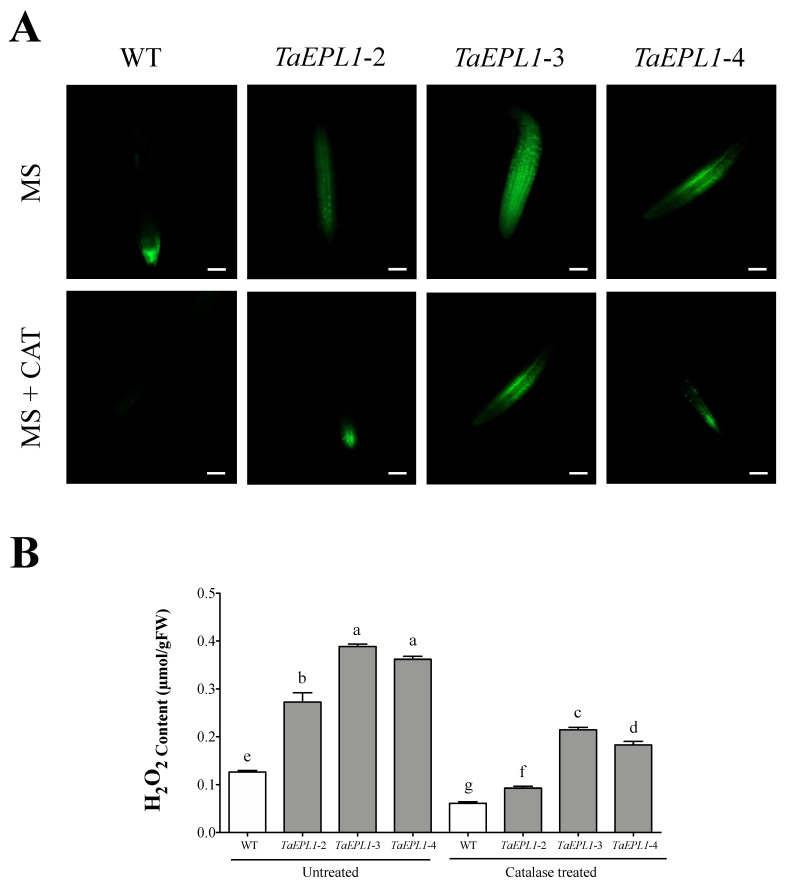
Detection of DCFH2-DA fluorescence in *Arabidopsis 35S::TaEPL1* lines and WT root tips. Representative images in root tips of ten-days-old WT and *35S::TaEPL1* seedlings (*TaEPL1-2*, *TaEPL1-3*, and *TaEPL1-4*). (**A**) Untreated (0.2X MS liquid medium) and catalase treated (0.2X MS + 250 U/mL CAT) root tips. Images were acquired on a Zeiss Axio Imager M2 microscope under 10× magnification. The scale bar corresponds to 100 μm. (**B**) H_2_O_2_ quantification was performed using KI in *A. thaliana* WT and *35S::TaEPL1* seedlings. Conditions shown are untreated (0.2X MS liquid medium) and catalase treated (0.2X MS + 250 U/mL CAT). The data show the means ± SE from three biological replicates (*n* = 3). The variance analysis was done by Two-way ANOVA (genotype x catalase), and the letters indicate statistically significant differences by Tukey’s test at *p* < 0.05.

In plants, ROS play an important role in plant defense against phytopathogen infection [30]. We analyzed whether the *35S::TaEPL1* lines produced a higher amount of ROS as a possible mechanism for resistance against microbes. We detected ROS in root tips of 10-day-old *35S::TaEPL1* and WT plantlets using 2,7-dichlorofluorescein diacetate (DCFH2-DA) dye and fluorescence microscopy. ROS signal, detected as green fluorescence, was more intense in *35S::TaEPL1* lines compared to WT roots (Figure 3A). Catalase (CAT) is a key antioxidant enzyme that alleviates oxidative stress through decomposition of hydrogen peroxides to water and oxygen. The CAT treatment was carried out to determine its effect on H_2_O_2_ levels between the *35S::TaEPL1* and WT lines. When we applied 250 U/mL CAT on *35S::TaEPL1* and WT plants, H_2_O_2_-associated fluorescence in the root tips in all transgenic lines was decreased, whereas in WT plants, no signal was detected (Figure 3A). A quantitative analysis of H_2_O_2_ confirmed that *35S::TaEPL1* lines accumulated a higher amount of hydrogen peroxide, with the highest levels observed in the *35S::TaEPL1*-3 line (3-fold more than WT) (Figure 3B). Moreover, upon the application of CAT enzyme (250 U/mL), both the transgenic *35S::TaEPL1* lines and the WT had decreased H_2_O_2_ levels, but the transgenic lines maintained the highest values (Figure 3B). 

### 2.5. The 35S::TaEPL1-3 Line Shows a Higher Accumulation of Transcripts of SA-Related Genes under Pseudomonas Syringae Infection

**Figure 4:**
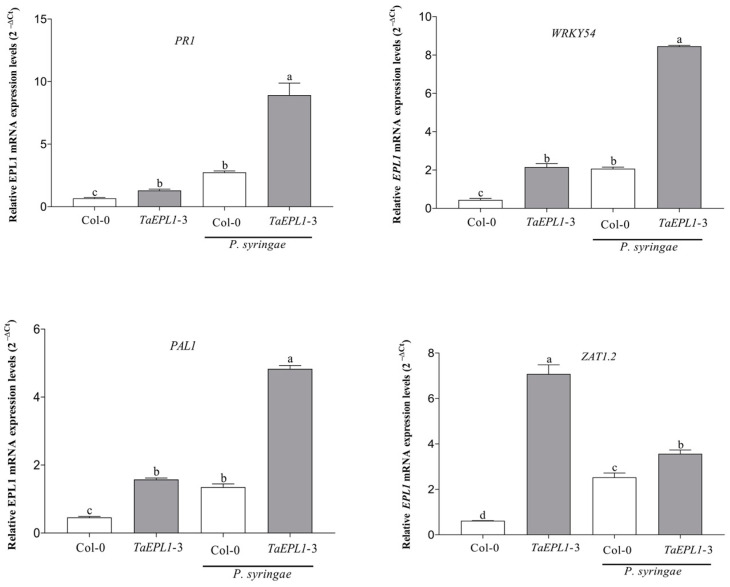
Expression analysis of plant defense genes in WT and *35S::TaEPL1-3* line during *Pseudomonas syringae* infection. Leaves of four-week-old WT and the *35S::TaEPL1-3* plants were infected or not with *P. syringae* for 24 h. The expression levels of *PR1*, *WRKY54*, *PAL1*, and *ZAT1*.2 genes were measured by RT-qPCR. Values were expressed as relative expression levels (2^−ΔCt^) calculated after normalization to the *A. thaliana UBQ5* gene. For each sample, three biological replicates were analyzed with their respective technical replicates. The variance analysis was done by Two-way ANOVA (genotype x infection), and the letters indicate statistically significant differences by Tukey’s test at *p* < 0.05.

Salicylic acid (SA) and reactive oxygen species (ROS) play important functions in the activation of plant defense under pathogen attacks. The expression levels of SA-mediated defense markers such as *PR1*, *PAL1*, and *WRKY54*, as well *ZAT1.2* (signaling ROS gene), were quantified by RT-qPCR in rosette leaves of 28-day-old WT and *35S::TaEPL1-3* plants inoculated with *P. syringae* (Figure 4). After 24 h of bacterial infection, we observed higher mRNA expression levels of the four genes analyzed (*PR1*, *PAL1*, *WRKY54* and *ZAT1.2*) in the *35S::TaEPL1-3* line compared to the WT (Figure 4). This same behavior was observed in the mock inoculated plants, in which all the measured markers showed a higher expression in the *35S::TaEPL1-3* line than the WT (Figure 4). This result is in accordance with the observation that this *EPL1* expressing line is also the most resistant to *P. syringae*. 

### 2.6. The Arabidopsis 35S::TaEPL1 Lines Were More Resistant to Botrytis cinerea Infection

**Figure 5:**
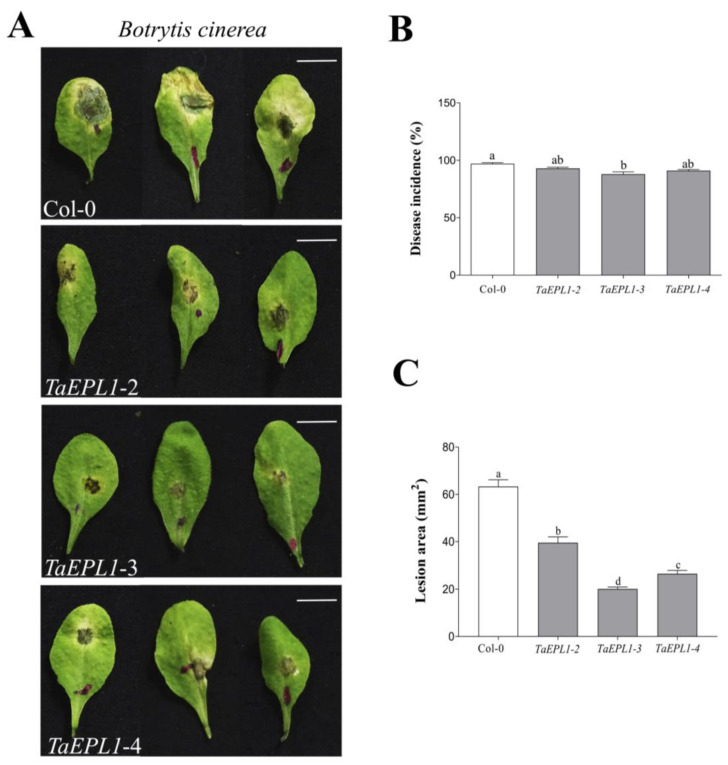
Leaf infection of *Arabidopsis 35S::TaEPL1* and WT by *Botrytis cinerea*. (**A**) Disease symptoms in leaves from four-week-old *35S::TaEPL1* (*TaEPL1-2*, *TaEPL1-3*, and *TaEPL1-4*) and WT plants at 72 h after infection with *B. cinerea* strain B05.10. For each line, three representative images of the infected leaves are shown. In the images, the scale bar indicates 1 cm. (**B**) The disease incidence graph shows the number of infection events with respect to the total inoculated leaves (*n* = 50) and expressed as a percentage. (**C**) Lesion area measurements (mm^2^) of total infection events. The bars denote the ± SE. Statistical analysis between genotypes was determined by One-way ANOVA, and the letters indicate statistically significant differences by Tukey’s test at *p* < 0.05.

**Figure 6:**
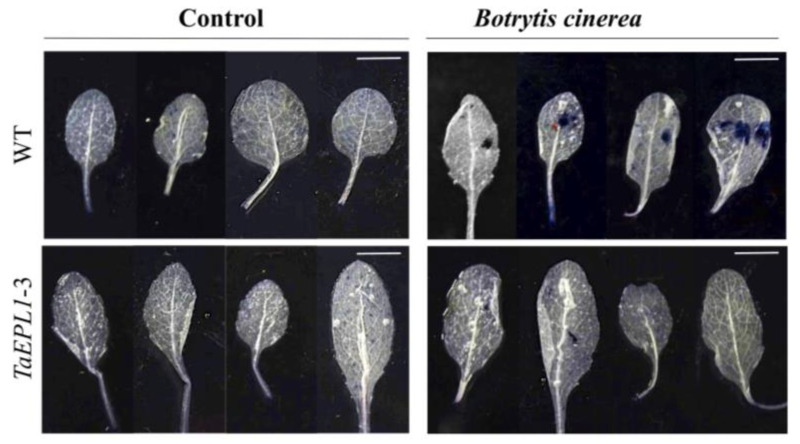
Trypan blue staining indicating cell death caused by *Botrytis cinerea* infection in leaves of *35S::TaEPL1-3* line and WT. Representative images of the area of cell death in *EPL1-3* and WT leaves inoculated with *B. cinerea*. In the images, the scale bar indicates 1 cm. Leaves of four-week-old WT and *EPL1-3* plants were infected with 2 × 10^5^ spores of *B. cinerea* strain B05.10. Leaves were collected 24 h after inoculation, and then stained in a trypan blue solution. As control, PDB (0.25X) was used as a mock treatment.

The resistance of *Arabidopsis* EPL1-expressing plants against infection by the necrotrophic fungus *B. cinerea* strain B05.10 was assessed. Four-week-old plants expressing *EPL1* gene (*EPL1-2*, *-3*, *-4*) were inoculated with the *B. cinerea* spore solution and compared with WT plants. After 72 hpi, the incidence of infection and leaf lesion size were evaluated. Figure 5A shows that the *35S::TaEPL1* expressing lines were more resistant to fungus infection than WT plants, as observed by decreased leaf necrosis and less water-soaking development. Necrotic lesion formation in the *35S::TaEPL1* lines showed reductions in a lesion size of 4.39 ± 0.1 mm^2^ for *EPL1-3*, 5.07 ± 0.1 mm^2^ for the *EPL1-4 line* and 6.13 ± 0.2 mm^2^ for the *EPL1-2* line, while the WT leaves had lesions of 7.77 ± 0.2 mm^2^ (Figure 5C). Even though disease incidence was not strongly reduced in the *EPL1* expressing lines, the *EPL1-3* line had the lowest necrotic lesion size caused by the fungus (Figure 5B), which agrees that this *EPL1* expressing line is the most resistant to the bacteria (Figure 2). In addition, we selected the *EPL1-3* line for trypan blue staining after inoculation with *B. cinerea* (Figure 6). We observed that the blue staining was lower in the *EPL1-3* line, while a strong signal was observed in the WT leaves (Figure 6). This confirms a decrease in plant cell death that correlates with a smaller lesion size in the transgenic *EPL1-3* line. 

### 2.7. Expression Changes Plant Defense Genes in the 35S::TaEPL1-3 Line under Botrytis cinerea Infection

**Figure 7:**
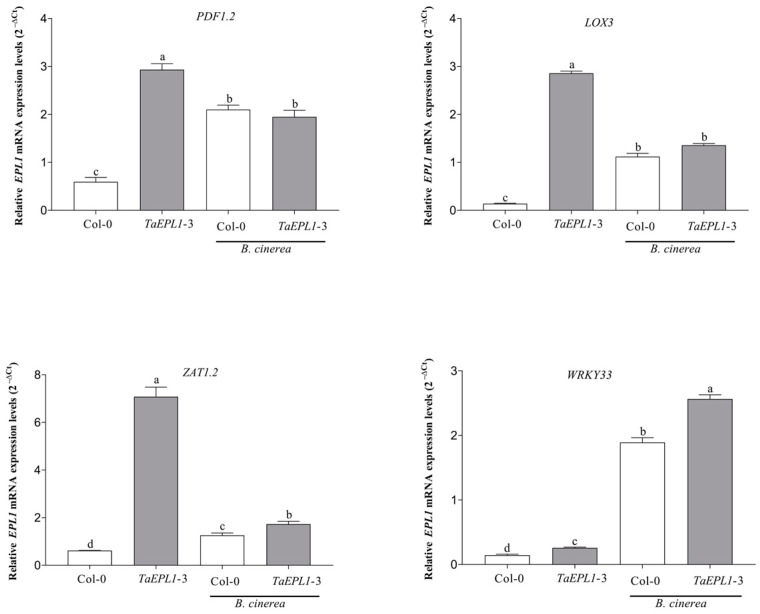
Expression analysis of plant defense genes in *A. thaliana* WT and *35S::TaEPL1-3* line during *Botrytis cinerea* infection. Leaves of four-week-old WT and the *35S::TaEPL1-3* plants were infected or not with fungus for 24 h. Expression patterns of *PDF1.2*, *LOX3*, *WRKY33*, and *ZAT1.2* genes of *A. thaliana* were assessed by RT-qPCR. Values were expressed as relative expression levels (2^−ΔCt^) calculated after normalization to the *A. thaliana UBQ5* gene. For each sample, three biological replicates were analyzed with their respective technical replicates. The variance analysis was done by Two-way ANOVA (genotype x infection), and the letters indicate statistically significant differences by Tukey’s test at *p* < 0.05.

We analyzed the expression levels of the *LOX3* and *PDF1*.2 genes involved in JA biosynthesis and response, respectively, *WRKY33* gene, which is a transcription factor implicated in plant defense against necrotrophic pathogens [31], and also *ZAT1.2* gene under *B. cinerea* infection. The gene expression levels were evaluated by RT-qPCR in rosette leaves of 28-day-old WT and *35S::TaEPL1-3* plants inoculated with *B. cinerea* for 24 h. In plants inoculated with the fungus, a higher expression was noticed in the *ZAT1.2* and *WRKY33* genes in the *35S::TaEPL1-3* line than WT; whereas, the *PDF1.2* and *LOX3* genes were similarly induced by the fungus between the WT and the *35S::TaEPL1-3* line (Figure 7). In the mock treatment, the *35S::TaEPL1-3* line had higher expression of all marker genes than the parental WT (Figure 7). Our data show that the *35S::TaEPL1-3* line has up-regulated plant defense genes against necrotrophic pathogens, which could prevent the spread of the fungus when plants were inoculated.

### 2.8. Determination of H_2_O_2_ Content in Arabidopsis WT and 35S::TaEPL1-3 Lines under B. cinerea and P. syringae Infection

**Figure 8:**
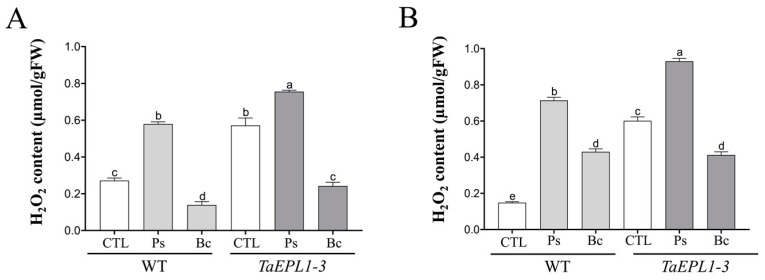
Hydrogen peroxide quantification in *A. thaliana* WT and *35S::TaEPL1-3* lines under *P. syringae* and *B. cinerea* infection. H_2_O_2_ quantification was performed using KI in *A. thaliana* 15 day-old WT and *35S::TaEPL1-3* plantlets. The data were represented graphically at 24 h (**A**) and 48 h (**B**) after the infection with *P. syringae* (Ps) or *B. cinerea* (Bc). The data show the means ± SE from three biological replicates (*n* = 3). The variance analysis was done by Two-way ANOVA (genotype x infection), and the letters indicate statistically significant differences by Tukey’s test at *p* < 0.05.

We analyzed the H_2_O_2_ accumulation in the 15 day-old WT and *35S::TaEPL1-3* plantlets under *B. cinerea* and *P. syringae* infection at 24 and 48 hpi (Figure 8). As observed in Figure 3, the *35S::TaEPL1-3* line accumulates a higher amount of ROS than WT under mock conditions (Figure 8). Despite the WT line showing an increase in H_2_O_2_ levels during bacterial infection at both 24 and 48 hpi, the *35S::TaEPL1-3* line exhibited the highest H_2_O_2_ accumulation (Figure 8). During *B. cinerea* infection, a decrease in H_2_O_2_ levels was observed at 24 and 48 hpi in the *35S::TaEPL1-3* line compared to their respective mock controls, whereas in the WT ecotype, there was a reduction in H_2_O_2_ levels at 24 hpi followed by an increase at 48 hpi compared to their mock controls (Figure 8).

## 3. Discussion

*Trichoderma* species are widely used in agriculture as a beneficial fungus due to its biocontrol activity and promotion of plant growth [1,32]. In the plant-Trichoderma interaction, both organisms secrete molecules for their recognition, initiating a molecular communication to achieve symbiosis between them [12,33,34,35,36,37,38,39]. Among the molecules reported to be secreted by Trichoderma are elicitors, such as Eliciting Plant Response-Like 1 (Epl1), which is produced and secreted by *T. atroviride*. There is evidence of a protective role of Epl1 protein from *T. atroviride* against foliar maize pathogen *C. graminicola* [21], and also strains of *T. atroviride* that overexpress *TaEPL1* gene increased resistance in tomato against *A. solani*, *B. cinerea*, and *P. syringae* infection [20]. Although the generation of *Arabidopsis* and rice plants expressing the SM1 elicitor of the phytopathogenic fungus *M. oryzae* has been reported, the effect of the expression in *A. thaliana* of an elicitor as EPL1 from beneficial fungus like Trichoderma and its resistance against pathogens has not been studied.

We describe here the response of *A. thaliana* plants that express the EPL1 elicitor to the infection by two phytopathogenic microorganisms. One of these pathogens was the hemibiotrophic *P. syringae*, in which *A. thaliana 35S::TaEPL1* lines were more resistant to bacterial infection. These *Arabidopsis 35S::TaEPL1* lines exhibited less damage caused by infection as well as lower bacterial load per leaf compared to the WT plants. This suggests that the EPL1 elicitor activates a defense response within the plant cells, thus generating a lower susceptibility to *P. syringae*.

We report that the expression of the EPL1 elicitor in *Arabidopsis* triggers H_2_O_2_ accumulation even in the absence of pathogen inoculation. Furthermore, despite applying CAT, a H_2_O_2_ scavenger, it did not completely reduce the H_2_O_2_-associated fluorescence signal in root tips of the transgenic lines, as observed in the WT. In this sense, the *35S::TaEPL1-3* line showed a higher *ZAT1.2* transcript level due to the infection of the bacterium as well as in the control plants (mock), which correlates with ROS accumulation in these transgenic lines. The *ZAT1.2* gene encoding a zinc finger protein is involved in oxidative stress responses [40]. After inoculation with *P. syringae*, the *TaEPL1-3* expression line had a higher accumulation of *PAL1*, *PR1*, and *WRKY54* transcripts, which are involved in the salicylic acid (SA) biosynthesis, response, and regulation, respectively. An interesting fact is that the highest accumulation of H_2_O_2_ was observed in the *35S::TaEPL1-3* line after bacterial infection, both at 24 and 48 hpi. The accumulation of ROS and the induction of these SA response genes could explain the resistance shown by the *35S::TaEPL1* lines to infection by Pseudomonas. It was previously reported that the plant protective activity of SM1 and other CP proteins are associated with the accumulation of ROS and phytoalexins [26,41,42].

Likewise, we found that the expression of the Ta*EPL1* gene in *Arabidopsis* confers enhanced resistance against *B. cinerea* infection. The *Arabidopsis 35S::TaEPL1* plants showed a smaller lesion area and a reduction of leaf cell death caused by the fungus compared to the WT plants. This is consistent with a higher expression of the *WRKY33* gene in the *35S::TaEPL1* line upon infection with the fungus *B. cinerea*. The WRKY33 transcription factor has been reported to be involved in defense responses against *B. cinerea* and other necrotrophic pathogens [31]. *TaEPL1* expression in *Arabidopsis* reduces fungal colonization of plant tissue, thus making them more resistant to this phytopathogen. An interesting observation was that the *LOX3*, *PDF1.2*, *WRKY33*, and *ZAT1.2* genes were up-regulated in the *35S::TaEPL1-3* line under mock conditions (i.e., no fungal infection), which could suggest that *EPL1* expression in the plant causes a priming of genes involved in plant defense against this necrotrophic pathogen. An important finding was that a reduction in H_2_O_2_ levels was observed in the *35S::TaEPL1-3* line during infection with *B. cinerea* at 24 and 48 hpi compared to the mock controls. This behavior suggests that the *EPL1* expressing line is actively regulating its ROS levels in response to the presence of this necrotrophic pathogen.

Our data provide evidence that *TaEPL1* expressed in *Arabidopsis* plants can activate defense responses against pathogens such as *P. syringae* and *B. cinerea*, which activate the SA- and JA/ET pathways. An ortholog to *EPL1* exists in *M. oryzae* called *MoSM1* [27]. Expression of *MoSM1* in rice significantly increased SA and JA content, and also induced SA- and JA-related biosynthesis genes and signaling under normal growth conditions [27]. In addition, the heterologous expression of the *MgSM1* in *A. thaliana* plants confers a broad-spectrum resistance against *B. cinerea*, *Alternaria brassicicola*, and *P. syringae* [26]. In the *MgSM1*-expressing plants, some defense genes such as *PR1*, *PR5*, and *PDF1.2* were upregulated, and an accumulation of ROS was reported [26]. The authors changed the 35S viral promoter to an inducible promoter, since large amounts of MgSM1 protein caused a hypersensitive response in *Arabidopsis*, while this phenotype has not been reported in the case of *T. virens* SM1 purified protein [18], nor in our study here with *TaEPL1*. We did not observe a visible hypersensitive response phenotype in any *35S::TaEPL1* lines. This could be due to the fact that *M. grisea* is a phytopathogenic fungus that causes severe disease in rice and other grasses, and the constitute expression of *SM1 in planta* generated a hypersensitive response with a greater accumulation of ROS, while species of the genus *Trichoderma* are plant symbionts, and so the higher expression of *EPL1* did not generate any negative phenotype in the plant. In addition to *SM1* from *M. grisea*, phytotoxic effects have been reported for cerato-platanin, such as BcSpl1 from *B. cinerea* [24], a CP of 12.4 kDa from *Ceratocystis fimbriata f. sp. platani* [28].

On the other hand, Arabidopsis plants carrying the *35S::TaEPL1* construct exhibited increased biomass, with the *TaEPL1-4* line showing the most pronounced accelerated growth. It is noteworthy that expressing a fungal elicitor, such as EPL1 (from the strain *T. atroviride* IMI 206040), in Arabidopsis results in the generation of more vigorous plants. Several species of Trichoderma have been reported to exert beneficial effects on plant growth [11]. In particular, the plant growth-promoting effect has been tested using the strain *T. atroviride* (IMI 206040) in tomato plants [20] and *A. thaliana* plants [12,32,34]. Further studies focused on the mechanism of *EPL1* gene are needed to elucidate its role in plant growth.

## 4. Materials and Methods

### 4.1. Plant Growth Conditions

Seeds of *Arabidopsis 35S::TaEPL1* lines generated in this work and parental Col-0 (WT) were sterilized using 20% (*v*/*v*) commercial sodium hypochlorite (6% free chlorine) solution for 5 min, and washed seven times in sterile distilled water. Aseptic seeds were stratified during 48 h at 4 °C and afterwards germinated and grown on agar plates containing 0.2X MS pH 7, 0.5% (*w*/*v*) sucrose, and 0.8% (*w*/*v*) agar. Seedlings were incubated in a growth chamber with a photoperiod of 16 h (100 μmolm^−2^ s^−1^)/8 h with a light/dark cycle at a temperature of 22 ± 1 °C until used in the experiments described above.

### 4.2. Generation of Arabidopsis thaliana 35S::TaEPL1 Lines

The EPL1 ORF (417 bp) was amplified from cDNA of mycelial tissue of the fungus *Trichoderma atroviride* with Pfu DNA Polymerase high fidelity (Thermo Scientific™, Waltham, MA, USA) using the primers: EPL1-Fw 5′ATGCAGTTCTCCAGCCTCTTCAAG3′ and EPL1-Rv 5′TTAGAGGCCGCAGTTGCTCACAGC3′. The product was cloned into the pCR8/GW/TOPO vector (Invitrogen, Carlsbad, CA, USA). The entry clone was verified by sequencing and recombined into pMDC32 binary vector by the Gateway LR Clonase enzyme mix (Invitrogen, Carlsbad, CA, USA) to generate the pMDC32-EPL1 construct. The pMDC32-EPL1 was transferred into *Agrobacterium tumefaciens* strain GV2260 by electroporation and transformed into *Arabidopsis thaliana* WT (Col-0) plants by the floral dip method [43]. Transformant seeds were selected based on their capacity to grow on 0.2X Murashige and Skoog medium (MS) supplemented with hygromycin at a concentration of 50 mg/mL. Three independent T4 *TaEPL1* overexpression lines were obtained and used in this work (*35S::TaEPL1-2*, *-3* and *-4*).

### 4.3. Measurement of Fresh Weight of 35S::TaEPL1 and WT Lines

*Arabidopsis thaliana* seeds of the WT (Col-0) and *35S::TaEPL1* expression lines (*TaEPL1-2*, *-3* and *-4*) were grown on 0.2X MS plates for 7 days. Subsequently, the seedlings were transferred to soil pots containing a mixture of Sunshine Mix #3 commercial substrate, perlite, and vermiculite (3:1:1). The pots were placed in a growth chamber with a temperature of 22 ± 1 °C with a photoperiod of 16 h (100 μmolm^−2^ s^−1^)/8 h, light/dark. The fresh weight (g) of the aerial part of each 40-day-old plant was measured using an analytical balance. Data analysis was done from six biological replicates (*n* = 6).

### 4.4. Pathogen Inocula Preparation

*Pseudomonas syringae* pv. Tomato DC3000 was grown to an OD600 nm of 0.8 in Luria Bertani (LB) medium (pH 7.0) supplemented with 50 mg/mL of rifampicin at 28 °C with shaking. The culture was centrifuged at 13,000 rpm for 10 min. The cells were washed twice with 10 mM MgCl_2_ and adjusted at an OD600 nm of 0.2 (1 × 10^8^ CFU/mL) for use in the infection assays. *Botrytis cinerea* strain B05.10 was grown on Potato Dextrose Agar (PDA) in darkness at 28 °C for two weeks. Spores were collected in sterile water, filtered through glass wool to remove hyphae, and quantified in a Neubauer chamber under a Motic model BA-300 microscope with 40× magnification (Motic^®^, San Antonio, TX, USA). The inoculum was diluted in Potato Dextrose Broth (PDB, 6 g/L) to a concentration of 1 × 10^6^ spores/mL^−1^.

### 4.5. Arabidopsis 35S::TaEPL1 Expression Lines—Pseudomonas syringae Inoculation Assays

Fourteen-day-old *35S::TaEPL1* and WT plants were transferred to 50 cell tray inserts (4.5 cm cell diameter) containing a sterile mixture of sunshine Mix#3 commercial substrate:vermiculite:perlite (3:1:1), and plants were watered every three days. Four-week-old plants were inoculated with *P. syringae* (50 plants of each line). For each plant, three leaves were infected with bacteria suspension at an OD600 nm of 0.2 in 10 mM MgCl_2_ using the abaxial injection method [44]. Mock inoculations of *35S::TaEPL1* and WT plants were made by infiltration with 10 mM MgCl_2_. Infiltrated plants were covered with a plastic dome to maintain humidity and incubated in a growth chamber with a photoperiod of 16 h (100 μmolm^−2^ s^−1^)/8 h with a light/dark cycle at a temperature of 22 ± 1 °C. For infection symptom evaluation, the leaves were collected 72 h post-inoculation (hpi), photographed, and sterilized using 70% ethanol for 1 min, and then rinsed with distilled water. Five independent 0.5 cm^2^ leaf disks from *35S::TaEPL1* and WT plants (*n* = 5) were ground in 10 mM MgCl_2_ and serially diluted. Ten microliters of a 1:5 dilution was spotted onto LB agar plates containing 50 mg/mL rifampicin, and colonies were counted after 2 days growth in the dark at 28 °C [44]. All interaction assays with bacteria were done three times with similar results.

### 4.6. Arabidopsis 35S::TaEPL1 Expression Lines—Botrytis cinerea Inoculation Assays

Plants of *35S::TaEPL1* and WT were cultivated and prepared for inoculation as described above. Four-week-old *35S::TaEPL1* lines and WT plants were inoculated with *B. cinerea* (50 plants of each line, *n* = 50). For each plant, three leaves were infected on the adaxial surface of leaves avoiding the vascular system with 10 μL drops containing 1 × 10^6^ spores. Control PDB (0.25X) was used as a mock treatment. Inoculated plants were covered with a plastic dome to maintain the humidity and placed in darkness for 72 h at a temperature of 22 ± 1 °C. After this period, the disease incidence (percentage of leaves that showed disease symptoms over the total number of inoculated leaves) and leaf lesion area using Image J software IJ 1.46r version (http://rsb.info.nih.gov/ accessed on 14 June 2023) were determined. Each assay was repeated three times with a similar result.

### 4.7. Plant Tissue Trypan Blue Staining after Botrytis cinerea Infection

Trypan blue dye was used to detect plant cell death after fungal infection. Infected leaves from four-week-old from *A. thaliana* WT and *EPL1*-3 plants were collected 24 hpi with 2 × 10^5^ spores of *B. cinerea* strain B05.10. PDB medium (0.25X) spore diluent was applied as a mock inoculation (control). Leaves were stained by immersing in a staining solution (10 mg of trypan blue, 10 mL of lactic acid, 10 mL of phenol, 10 mL of glycerol, and 10 mL of distilled H_2_O) for 12 h. Leaves were cleared with consecutive washes in ethanol (98%) until chlorophyll was completely removed and photographed.

### 4.8. Hydrogen Peroxide (H_2_O_2_) Detection by DCFH_2_-DA Labeling

In situ localization of reactive oxygen species (ROS) was performed in root tips of ten-day-old WT and *35S::TaEPL1* lines using the fluorescent molecule 2′,7′-dichlorodihydrofluorescein diacetate (DCFH2-DA, Sigma-Aldrich, Burlington, MA, USA), as described by [45], with some modifications. The effect of the exogenous application of catalase (CAT, Sigma Aldrich, Burlington, MA, USA) on the H_2_O_2_ levels in the WT and *35S::TaEPL1* lines was also analyzed. Seedlings were placed in 24-well culture plates containing 0.2X MS liquid supplemented with 0 or 250 U/mL of CAT (dissolved in pH7 phosphate buffer) and incubated at 22 ± 1 °C in a growth chamber for 4 h with continuous light. The seedlings were then immersed in 25 μmol DCFH_2_-DA in Tris buffer (10 mM Tris, 50 mM KCl, pH 7.2) for 30 min in complete darkness. After rinsing with buffer to remove excess DCFH_2_-DA and CAT, the roots were observed and photographed with a fluorescence microscope (Zeiss Axio Imager M2; Carl Zeiss Microscopy, Pleasanton, CA, USA) at 10× magnification using excitation and emission wavelengths of 480 nm and 500–550 nm, respectively. Fifteen seedlings of WT and *35S::TaEPL1* lines were analyzed by treatment; each assay was repeated at least three times.

### 4.9. Determination of Hydrogen Peroxide Content with Potassium Iodide (KI)

Hydrogen peroxide content was determined according to Jungle et al., 2014. Ten-day-old plantlets of WT and *35S::TaEPL1* lines were transferred to plates containing 0.2X MS liquid supplemented with 0 or 250 U/mL of CAT and incubated at 22 ± 1 °C in a growth chamber for 4 h with continuous light. The CAT treatment was carried out to determine the reduction of the H_2_O_2_ content between the WT and *35S::TaEPL1* lines. In another experiment, we measured the hydrogen peroxide content in 15-day-old *A. thaliana* WT and *35S::TaEPL1-3* plants following inoculation with pathogens. Plantlets were infected with 2 μL of *P. syringae* or *B. cinerea* inocula for 24 h and 48 h, and as mock treatments, MgCl_2_ and PDB (0.25X) were used, respectively. Samples of 100 mg of fresh tissue from whole *Arabidopsis* plantlets were homogenized in an ice bath with 375 μL 0.1% (*w*/*v*) trichloroacetic acid. The mixture was centrifuged at 4 °C, 7000 rpm for 20 min; 250 μL of supernatant was transferred to a new tube and 250 μL of 10 mM potassium phosphate buffer (pH 7.0) was added, followed by 500 μL of 1 M KI. The absorbance was read at 390 nm using an Epoch-2 microplate reader (Biotek^®^, Winooski, VT, USA). The H_2_O_2_ content was determined using a standard curve (10, 20, 30, 50, 70, and 100 μM H_2_O_2_). Data analysis was done from three biological replicates (*n* = 3).

### 4.10. RNA Isolation and RT-qPCR Gene Expression Analysis

Total RNA was extracted from 10-day-old seedlings of WT and *35::EPL1-2*, *-3*, and *-4* lines using Concert reagent (Invitrogen, Carlsbad, CA, USA), followed by DNAase Turbo digestion (Ambion, Austin, TX, USA) for genomic DNA removal. Total RNA concentration was calculated with an Epoch-2 microplate reader (Biotek^®^ Winooski, VT, USA). cDNA was synthesized using 1 μg total RNA and a SuperScript™ II Reverse Transcriptase kit (Invitrogen, Carlsbad, CA, USA). RT-qPCR was performed using the Step One Real-Time PCR Detection System (Applied Biosystems, Waltham, MA, USA). The reaction mixture was made in a volume of 10 μL containing 100 ng of cDNA, 200 nM of each primer (Supplementary Table S1), and 5 μL of Maxima SYBR Green/ROX qPCR Master Mix (2×) Thermo Scientific™ (Waltham, MA, USA). First, the relative expression of the ELP1 gene in *35S::TaEPL1* Arabidopsis lines was quantified. Then, the *EPL1-3* line was selected to measure the expression levels of SA-mediated defense markers, such as *PR1*, *PAL1*, and *WRKY54* and JA-mediated defense markers *LOX3* and *PDF1.2*, *WRKY33*, as well *ZAT1.2* (signaling ROS gene). Expression levels were quantified in rosette leaves of 28-day-old WT and *35S::TaEPL1-3* plants inoculated with *B. cinerea* or *P. syringae* for 24 h. The sequences of oligonucleotides used are included in the Supplementary Table S1. Gene expression was analyzed by the delta Ct method [46]. The *AtUBQ5* gene was used as a reference. For each cDNA sample, three biological replicates (*n* = 3) were analyzed with their respective technical replicates.

### 4.11. Statistical Analysis

Results from representative experiments are shown as means ± SE. Statistical significance (*p* ≤ 0.05) among data were determined regarding genotypes by One-Way ANOVA or Two-Way ANOVA (genotype x infection). Tukey’s post-tests were performed using the GraphPad Prism version 8.0 software (GraphPad Software, San Diego, CA, USA).

## 5. Conclusions

Our data show that *Arabidopsis* lines expressing the *EPL1* elicitor of *T. atroviride* are clearly resistant to pathogens with different lifestyles and modes of nutrition, namely, the hemibiotrophic bacteria *P. syringae* and the necrotrophic fungus *B. cinerea*. We observed that the *Arabidopsis EPL1-3* line, which had the highest expression of the Ta*EPL1* gene, resulted in greater protection against pathogens. This reduction in pathogen infection in *EPL1* expressing plants could be correlated with the increase in ROS levels and the induction of SA- and JA-related genes. Therefore, EPL1 elicitor is an excellent candidate for use as a molecule that provides protection against diverse phytopathogens.

## Data Availability

The data presented in this study are available in the manuscript.

## Supplementary Materials

The following supporting information can be downloaded at: https://www.mdpi.com/article/10.3390/plants12132443/s1, Figure S1: Generation of *35S::TaEPL1* Arabidopsis lines. Table S1: Oligonucleotide sequences used to measure gene expression by RT-qPCR analysis.
