# Supplementary material for: Expression of EPL1 from Trichoderma atroviride in Arabidopsis Confers Resistance to Bacterial and Fungal Pathogens
Source: Plants (Basel). 2023 Jun 25;12(13):2443. doi: 10.3390/plants12132443 (PMC10347261; doi:10.3390/plants12132443)
Supplement: Supplementary file 1 [file plants-12-02443-s001.zip › plants-2386834-supplementary.pdf]

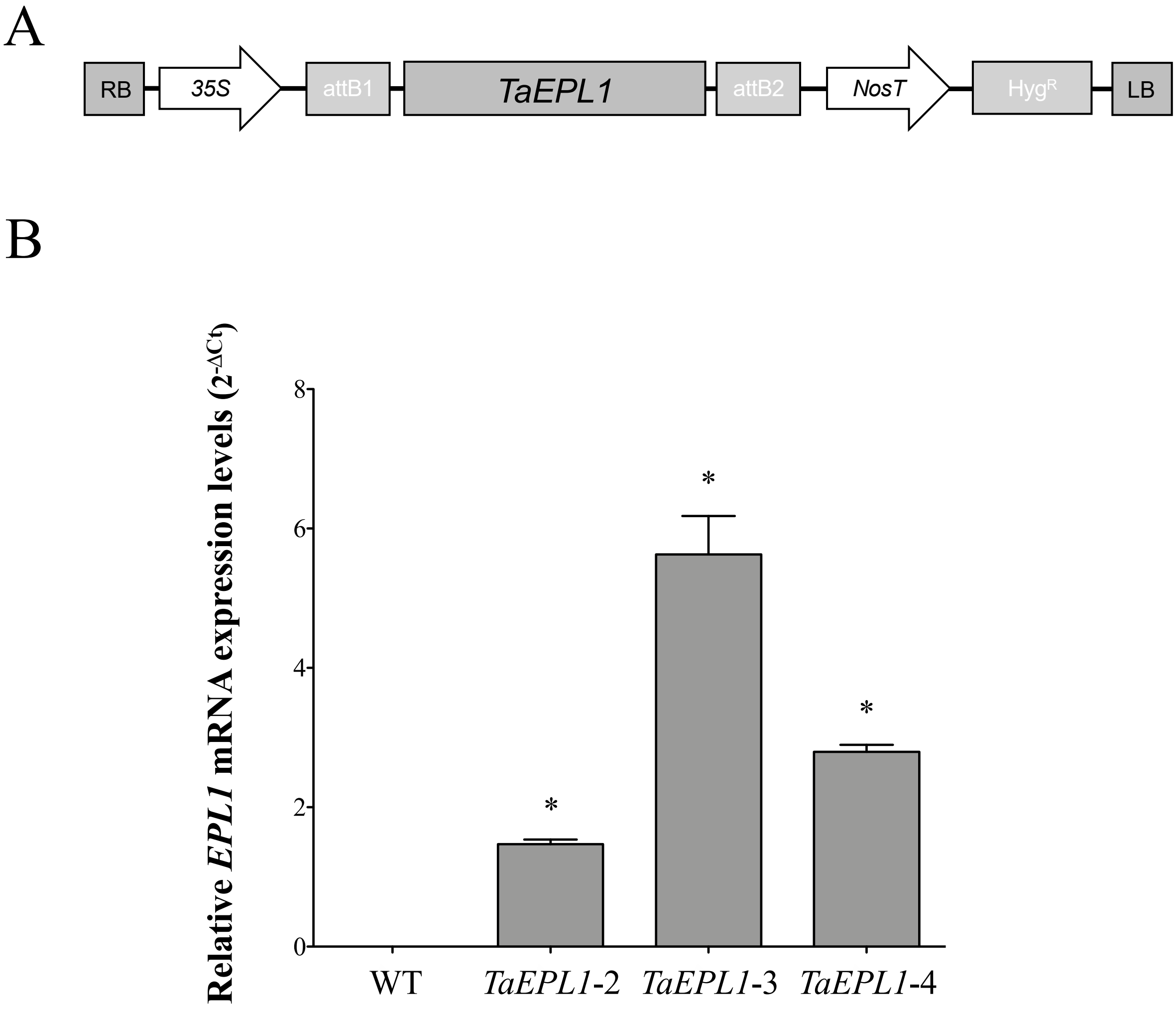

Supplementary Figure S1. **Generation of *35S::TaEPL1* Arabidopsis lines. A**. Schematic representation of the *35S::TaEPL1* construct in pMDC32 binary vector. RB and LB borders for T-DNA integration; 35S, cauliflower mosaic virus 35S promoter; attB1 and attB2 sites for recombination; *TaEPL1*, *T. atroviride EPL1* cDNA; *Nos T*, nopaline synthase terminator region; Hyg^R, *HPTII* hygromycin resistance gene. **B**. Expression level of *TaEPL1* gene was measured in fifteen days old *35S::TaEPL1* (*TaEPL1-2*, *TaEPL1-3* and *TaEPL1-4*) and WT plantlets by RT-qPCR. Quantification was expressed as relative expression levels ($2^{-\Delta Ct}$) calculated after normalization to the *A. thaliana UBQ5* gene.

Supplementary Table S1. Oligonucleotide sequences used to measure gene expression by RT-qPCR analysis

Locus	Gene	Sequences
AT3G62250	*UBQ5*	Fw-GGAGTGCCCTAACGCAACC<br>Rv-GCTACAACAGATCAAGCTTCAAC
AT2G37040	*PAL1*	Fw- GTGGCTTGTTTCTTTCGTGCTT<br>Rv-TGGTGTTACTACTGGTTTTGGTGCT
AT1G17420	*LOX3*	Fw-CTTCACTGCTGGTGCATACG<br>Rv-AAGACCATGTGGTTGTGTTGCA
AT2G46800	*ZAT1.2*	Fw-ATCAAGTCGACGGTGGATGT<br>Rv-ACAAAGCGTCGTTGTTAGGC
AT2G40750	*WRKY54*	Fw-GGTTTGGCAAGAGACGATGAT<br>Rv-CATACTCAAAGAGAAGACCTAG
AT2G14610	*PR1*	Fw-TTCTTCCCTCGAAAGCTCAA<br>Rv-AAGGCCCACCAGAGTGTATG
AT5G44420	*PDF1.2*	Fw-ATGGCTTAAGTTTGCTTCCAT<br>Rv-TTAACATGGGACGTAACAGA
	TaEPL1	Fw-ATGCAGTTCTCCAGCCTCTTCAAG<br>Rv-GTGTGGATGAGTTCATGTTAGC
